# Reliability Estimation of Parameters of Helical Wind Turbine with Vertical Axis

**DOI:** 10.1155/2015/296762

**Published:** 2015-06-18

**Authors:** Adela-Eliza Dumitrascu, Badea Lepadatescu, Dorin-Ion Dumitrascu, Anisor Nedelcu, Doina Valentina Ciobanu

**Affiliations:** ^1^Department of Manufacturing Engineering, Transilvania University of Brasov, 29 Eroilor Street, 500036 Brasov, Romania; ^2^Department of Automotive and Transport Engineering, Transilvania University of Brasov, 1 Politehnicii Street, 500024 Brasov, Romania; ^3^Department of Forest Engineering, Forest Management and Terrestrial Measurements, Transilvania University of Brasov, 1 Sirul Beethoven Street, 500123 Brasov, Romania

## Abstract

Due to the prolonged use of wind turbines they must be characterized by high reliability. This can be achieved through a rigorous design, appropriate simulation and testing, and proper construction. The reliability prediction and analysis of these systems will lead to identifying the critical components, increasing the operating time, minimizing failure rate, and minimizing maintenance costs. To estimate the produced energy by the wind turbine, an evaluation approach based on the Monte Carlo simulation model is developed which enables us to estimate the probability of minimum and maximum parameters. In our simulation process we used triangular distributions. The analysis of simulation results has been focused on the interpretation of the relative frequency histograms and cumulative distribution curve (ogive diagram), which indicates the probability of obtaining the daily or annual energy output depending on wind speed. The experimental researches consist in estimation of the reliability and unreliability functions and hazard rate of the helical vertical axis wind turbine designed and patented to climatic conditions for Romanian regions. Also, the variation of power produced for different wind speeds, the Weibull distribution of wind probability, and the power generated were determined. The analysis of experimental results indicates that this type of wind turbine is efficient at low wind speed.

## 1. Introduction

Environmental pollution, greenhouses effect, and intensification due to the wide ranging of organic fuel, as well as care of energy supply for the future generations, caused the acceleration of the development of technologies oriented to the implementation of renewable energy sources.

As the global fuel price increases these days, power systems using renewable energy sources have attracted attention. Moreover, environmental issues are accelerating change into alternative power systems [1]. Environmental concerns are growing, interest in environmental issues is increasing, and the idea of generating electricity with less pollution is becoming more and more attractive. Unlike conventional generation systems, “fuel” for solar photovoltaic energy and wind energy is available at no cost.

Wind power is today the fastest growing electricity generation technology. Impressive annual growth rates of more than 35% between 2001 and 2010 have made Europe into a frontrunner in wind energy technology development.

Wind energy is recognized worldwide as a proven technology to meet increasing electricity demand in a sustainable and clean manner. Offshore wind energy has the added attraction that it has minimal environmental impact and less noise problems [2].

All around the world there are a number of small isolated communities, like island and rural villages without access to a large electricity grid. Further, in many places, due to remoteness and cost, it is unlikely that a main grid connection will ever be established, as in remote telecommunications stations, research centers, and so forth.

Estimates show that a significant number of isolated consumers are spread all over Europe, including mainly country houses, inaccessible farms, shelters, telecommunications stations, small islands, and light houses. In Romania, a large number of remote consumers possess an outstanding wind potential. Unfortunately, the absence of an electrical network in their major area or the prohibitively high consumers cost—due to large distances and peculiar topography—forces them to cover their urgent electrification needs by using small diesel electric generators, with all the known problems of pollution of environment and noise.

In this respect, an alternative can be the wind turbine.

Wind turbine systems are rapidly becoming an economically viable source of renewable energy. Two key elements of wind turbine technology are turbine performance and availability. Turbine performance-energy produced is a function of design variables and a highly stochastic operating environment. Machine availability is a function of system reliability, and it is impacted by the design, the operating environment, and the maintenance considerations [3].

Most wind turbine manufacturers include failure analysis as an essential part of their continuous quality improvement process. Evaluating the root cause of a major component failure is essential to determining if the failure is due to manufacturing quality, product misapplication, design error, or inappropriate design assumptions [4].

The traditional method of measuring wind turbine performance under laboratory conditions in ideal circumstances will always tend to be optimistic and rarely reflect how the turbine actually behaves in a real situation—local wind conditions, nearby obstructions, power demand, and so forth. There will also be deterioration in performance with time due to wear and tear. What is important is how the turbine reacts and actually delivers power on site [5]. In the design of the various functional elements of a wind turbine, blade design is given the highest priority. Therefore, in many respects, the aerodynamic and dynamic properties of the blade decisively influence the entire system. The capability of the rotor drive to convert a maximum proportion of wind energy into mechanical energy for blade rotation is obviously the direct result of its aerodynamic properties. These features, in turn, largely determine the overall efficiency of energy conversion in the wind turbine [6, 7].

Analyzing the specialized literature in this field, the main concerns in the development of horizontal axis wind turbines were found. In this way, the purpose of the paper is to estimate the performance, advantages, and areas of usage with high efficiency of the vertical axis wind turbines.

The main advantages of this type of wind turbine with helical rotor, in which main rotor shaft runs to the flow streamlines, over other types of wind turbines with horizontal axis are as follows:

- it has high reliability;
- in an isolated area, with no connection to the national network, a helical type vertical axis wind turbine can be used with good results;
- it has simplicity in construction and good rigidity;
- it has smaller cost with 20% as similar turbines;
- it has specific power bigger on the active surface;
- it has high torque moment at starting;
- at wind speeds greater than 20 m/s there is self-braking without mechanical components, due to its original shape of rotor;
- it does not need orientation into the wind direction;
- it can work to high wind speed, 50 m/s;
- it is the only wind turbine that is accepted by environmental agencies, because it does not kill birds;
- it does not make noise during its operation.

Our research in this field began about ten years ago; the partial results of our research were focusing on the design and testing in operation [8]. In this paper, the investigations were extended with original elements, especially with reference to reliability estimation based on technological and construction parameters of helical vertical axis wind turbine.

## 2. Materials and Methods

### 2.1. Characteristics of Designed Wind Turbine

The helical vertical axis wind turbine will be analyzed and tested for Romanian regions in order to estimate the reliability (unreliability) function and hazard rate; it is designed to generate 1000 W.

This type of wind turbine is easy to mount on the roof of a house, having the main advantage that it does not need to be pointed into the wind direction with a system as with other types of wind turbines. At the same time it works without any noise and the shape of the rotor makes these turbines less likely to damage birds.

**Figure 1:**
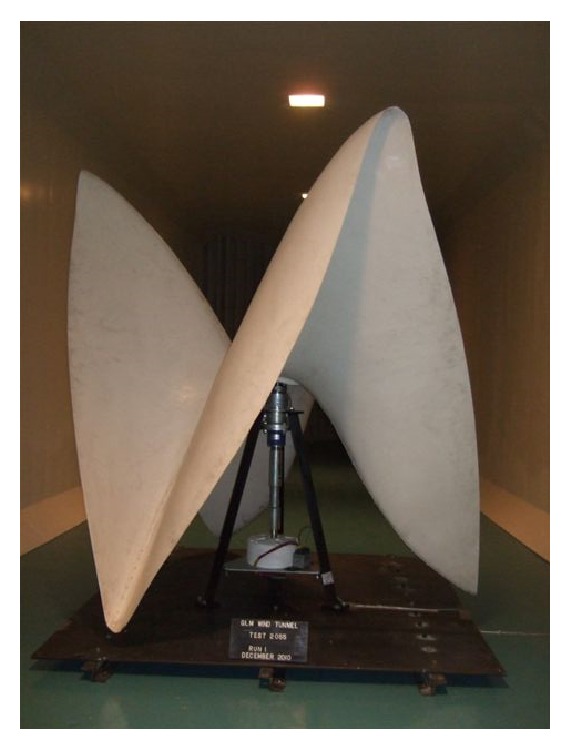
Helical rotor shape tested in aerodynamic tunnel.

The rotor of this wind turbine was made by the FINEX Company, patented in Romania, with a special shape helical type and manufactured from fiber glass material (Figure 1) [8].

The rotor was mathematically designed and tested in experimental conditions on the car adapted as a mobile laboratory, where the following parameters were measured:

- power of generator (W);
- wind speed (m/s);
- spindle revolutions (rpm);
- air temperature (°C).

Because the rotational speed of the rotor shaft is a maximum of 120 rpm, a device was needed that increased the rotational speed and ensured optimum working conditions for the permanent generator magnet. The electric current is three-phased and a device was needed to modify it in order to measure with as good an accuracy as possible the current generated at different rotor shaft rotational speeds.

Also, in Table 1 are specified the technical characteristics of wind turbine with vertical axis for a power of 1000 W.

After the experimental tests, it results in the next formulae of calculation of the power generated by the wind turbine with vertical axis helical type:

(1) $$\begin{array}{c} P=k\times A\times v^{3}, \end{array}$$

where *k* is the power coefficient, *A* is the rotor surface (m^2^), and *v* is the wind speed (m/s).

One rotor can generate a power of 600 W, at a wind speed of 12 m/s, and by mounting on a common axle of two rotors it produces a total power of 1200 W.

### 2.2. Monte Carlo Simulation of Parameters of Helical Wind Turbine with Vertical Axis

The Monte Carlo simulation of parameters of helical vertical axis wind turbine includes elements of reliability theory and notions of probability and statistical inferences.

Since the beginning of the 1970s, an intensive development of the use of Monte Carlo method has taken place, due to a massive increase of the new generations computers' processing power. At the same time, the formalism linked with the Monte Carlo method application has been intensively developed [9–11].

Particular role in learning the reality could be attributed to the methods using occurrences treated as random, but only a development of sciences associated with random processes provided rational basis to evaluate methods and the results of examining these processes. It turned out that rational treating of the occurrences, whose natures—as random—are essentially irrational, can be the source of useful knowledge about objectively learned reality. Formalized approach to learning the reality—thanks to the random reality created by people—found its place in science under the name of Monte Carlo method, which can be associated not only with a world's hazard capital, but also—what is important—with elegance, as the Monte Carlo method itself, and eternal longing of people for power, either material or intellectual [12].

In the Monte Carlo method, calculations are repeated several times using the same deterministic model of a physical phenomenon but each time for different, randomly selected values of particular arguments, from among uncertainty range given a priority. The Monte Carlo technique is a device for modeling and simulating processes that involve chance variables [13, 14].

By studying the distributions of results, we can see the range of possible outcomes and the most likely results. Using simulation, a deterministic value can become a stochastic variable. We can then study the impact of changes in the variable on the rest of the spreadsheet.

The recent popularity of the triangular distribution can be attributed to its use in the Monte Carlo simulation modeling and its use in standard uncertainty analysis software. The triangular distribution is also found in cases where two uniformly distributed errors with the same mean and bounding limits are combined linearly [15].

Uncertainties may be modeled by the distribution where the authors discuss the asymmetric triangular distribution [16, 17]. Suppose that

(2) $$\begin{array}{c} x_{i}=\left\{\begin{array}{cc} a+\sqrt{z_{i}\cdot \left(b-a\right)\cdot \left(m-a\right)}, & a<x_{i}\leq m,\\ b-\sqrt{\left(1-z_{i}\right)\cdot \left(b-a\right)\cdot \left(b-m\right)}, & m<x_{i}\leq b, \end{array}\right. \end{array}$$

where $\hat{a}$ is lower estimate, $\hat{m}$ is most likely estimate value, and $\hat{b}$ is maximum estimate value.

The mean and standard deviation are given by

(3) $$\begin{array}{c} \mu =\frac{a+m+b}{3},\\ \sigma =\sqrt{\frac{a^{2}+m^{2}+b^{2}-am-ab-mb}{18}}. \end{array}$$

The distribution emerges in numerous papers and the probability density function for asymmetric three-parameter is given by [16, 17]. Consider

(4) $$\begin{array}{c} f\left(x\right)=\left\{\begin{array}{cc} \frac{2\left(x-a\right)}{\left(b-a\right)\cdot \left(m-a\right)}, & a<x\leq m,\\ \frac{2\left(b-x\right)}{\left(b-a\right)\cdot \left(b-m\right)}, & m<x<b,\\ 0, & \text{elsewhere}. \end{array}\right. \end{array}$$

Cumulative distribution function is defined by

(5) $$\begin{array}{c} F\left(x\right)=\left\{\begin{array}{cc} 0, & x\leq a,\\ \frac{{\left(x-a\right)}^{2}}{\left(b-a\right)\cdot \left(m-a\right)}, & a<x\leq m,\\ 1-\frac{\left(b-x\right)}{\left(b-a\right)\cdot \left(b-m\right)}, & m<x<b,\\ 1, & x\geq b. \end{array}\right. \end{array}$$

**Figure 2:**
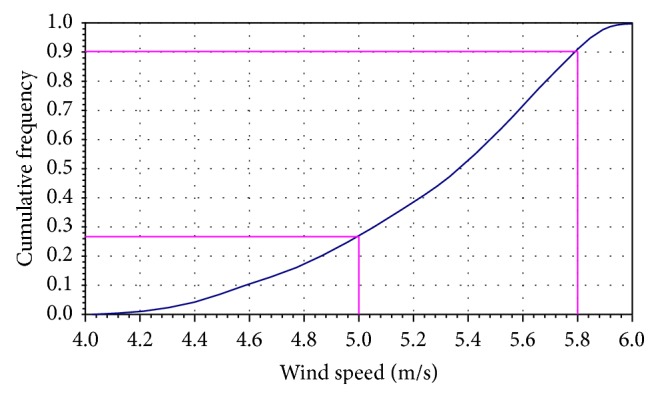
Cumulative frequency distribution for wind speed using Monte Carlo simulation.

Cumulative distributions functions are usually presented graphically in the form of ogives, where we plot the cumulative frequencies at the class boundaries. The resulting points are connected by means of straight lines, as shown in Figures 2, 4, and 5.

If the relative frequency is plotted on normal probability graph paper, the ogive will be a straight line for a normally distributed random variable. The normal probability graph paper is a useful device for checking whether the observations come from a normally distributed population, but such a device is approximate. One usually rejects normality when remarkable departure from linearity is quite evident.

The Monte Carlo simulation of parameters of helical vertical axis wind turbines includes elements of reliability theory and notions of probability and statistical inferences. In our simulation process we used triangular distributions because the input data can be obtained very easily and it does not require laborious investigations.

The most important parameter that influences the reliability of vertical axis wind turbines is wind speed. In this case, interesting results may be expected from the implementation of the Monte Carlo simulation. Considering the wind speed between 4 m/s and 6 m/s for Romanian analyzed region, the cumulative probability can be determined and expressed by the cumulative frequency curve (ogive chart) for this data and looks like in Figure 2.

**Figure 3:**
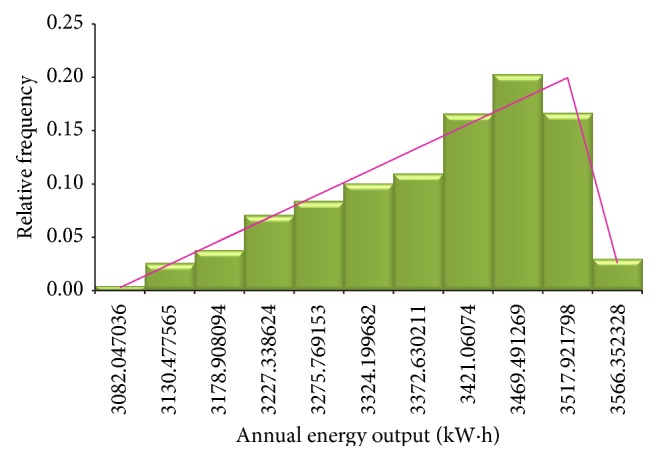
Monte Carlo simulation of relative frequency histogram for annual energy output.

**Figure 4:**
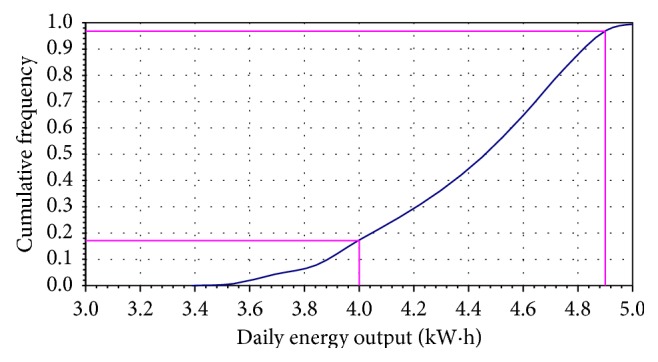
Monte Carlo simulation of cumulative frequency distribution for daily energy output.

Another measure of helical wind turbine reliability is the annual energy production. In a similar manner, the daily and annual energy output can be simulated. As a result, the simulation data are represented by relative frequency histograms (Figure 3) and ogive diagram (Figure 4). The cumulative distribution curve indicates the probability that the daily or annual energy output will be obtained. Each time when the simulation is executed, the cell will be updated to show a random value drawn from the specified distribution.

**Figure 5:**
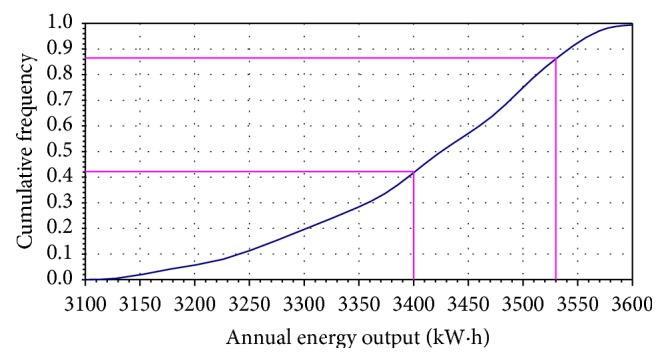
Monte Carlo simulation of cumulative frequency distribution for annual energy output.

Figure 5 shows the simulation results of annual energy output according to relative frequency histograms, and the standard asymmetric triangular distribution can be seen. Analyzing the cumulative frequency distribution (Figures 2, 4, and 5), the simulation results are presented synthetically in Table 2.

It can be observed that the probability to exceed the maximum value of wind turbine parameters is approximately 9.6% for wind speed, 3% for daily energy, and 11% for annual energy. These percentages are obtained for different intervals set as acceptable limits.

### 2.3. Reliability Estimation of Helical Wind Turbine with Vertical Axis

Reliability is considered to be a performance attribute that is concerned with the probability of success and frequency of failures and is defined as “The probability that an item will perform its intended function under stated conditions, for either a specified interval or over its useful life” [18–20].

The reliability estimation of the helical vertical axis wind turbine type is focused on collecting real-time power, rotor speed, and wind speed data of a specific region from Romania. Also, the wind turbine has operated for 5 years.

The Weibull distribution is a powerful modeling tool used in reliability analyses to predict failure rates and to provide a description of the failure of parts and equipment. The Weibull distribution is one of the most widely used distributions in reliability calculations. The great versatility of the Weibull distribution stems from the possibility to adjust to fit many cases where the hazard rate either increases or decreases. Further, of all statistical distributions that are available the Weibull distribution can be regarded as the most valuable because through the appropriate choice of parameters (the location parameter, the shape parameter, and the scale parameter), a variety of shapes of probability density function can be modeled [16, 17, 20–25].

The measurements of wind speed (mean and standard deviation) are made periodically, and the resulting wind speed data is modeled as a Weibull distribution with probability density function (PDF) as shown in the following equation [16, 17, 20–25]:

(6) $$\begin{array}{c} f\left(v\right)=\frac{\beta }{\eta }\cdot {\left(\frac{v}{\eta }\right)}^{\beta -1}\cdot \exp\left[-{\left(\frac{v}{\eta }\right)}^{\beta }\right], \end{array}$$

where *f*(*v*) is the probability density function of the wind speed distribution, *v* is the wind speed, *β* is the Weibull shape parameter, and *η* is the scale parameter.

**Figure 6:**
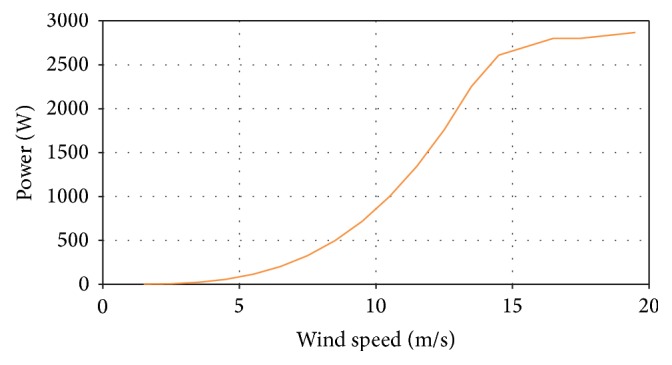
Power diagram variation at different wind speeds for analyzed Romanian regions.

Based on experimental tests, the variation of power produced for different wind speeds is illustrated in Figure 6. The cut-in wind speed is 1.8 m/s, the cut-out wind speed is 20 m/s, and the rated power is 2900 Watts (W).

The statistical processing of experimental data allows us to determine the statistical parameters of distribution and reliability indices using specialized functions from Excel, MathCAD, and Weibull++ software in order to estimate the reliability and unreliability functions, probability density function, and hazard rate (failure rate) of the helical vertical axis wind turbine.

Assuming that the time to failure of the wind turbine follows the Weibull distribution with test time expressed in years. The probability of a failure during the first five years is

(7) $$\begin{array}{c} \mathrm{Pr}\left(t\leq 5\right)=1-\exp\left[-{\left(\frac{t}{\eta }\right)}^{\beta }\right]=0.00298. \end{array}$$

**Figure 7:**
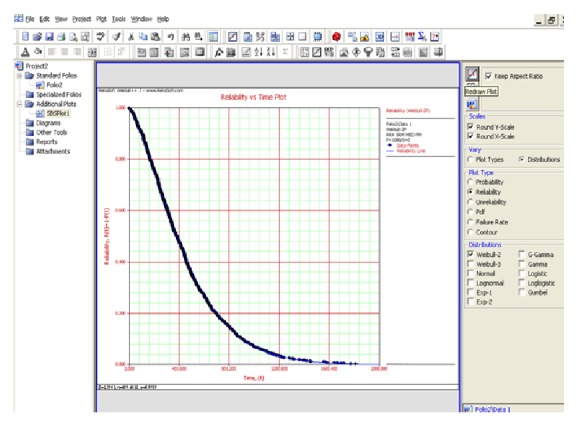
Reliability estimation.

**Figure 8:**
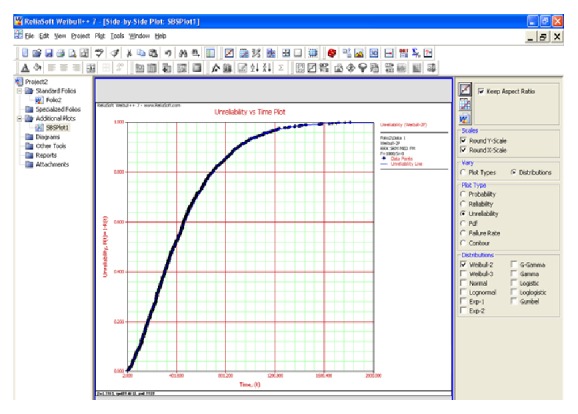
Unreliability estimation.

The life distribution of each parameter that influences the wind turbine reliability is assumed to be a standard two-parameter Weibull distribution. Using the Weibull distribution, a more accurate failure distribution can be calculated, which takes into account the weather, temperature, and power variations that drive turbine life. To estimate the reliability and unreliability function of helical wind turbine, a simulation approach was made (Figures 7 and 8).

## 3. Results and Discussions

The real performance of the vertical axis wind turbine will be affected by local wind conditions, nearby obstructions, power demand profiles, and a range of other factors.

The results of experimental tests were verified using Kolmogorov-Smirnov test in order to decide if a sample comes from a population with a specific distribution. The validation of statistical model consisted of calculation of the empirical distribution function of the sample and the cumulative distribution function of assumed Weibull distribution. The result was compared with a confidence level of *α* = 0.05. After the implementation of the Kolmogorov-Smirnov test, the Weibull distribution was accepted.

**Figure 9:**
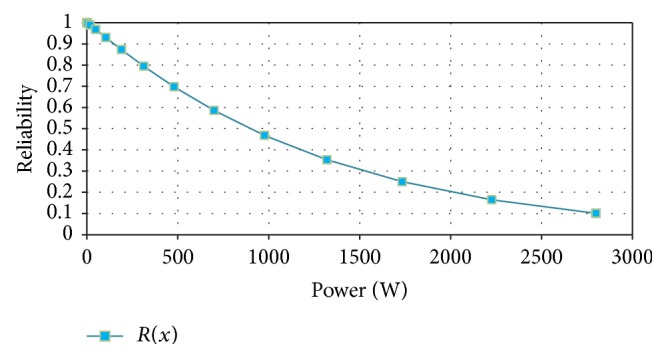
Reliability versus power.

**Figure 10:**
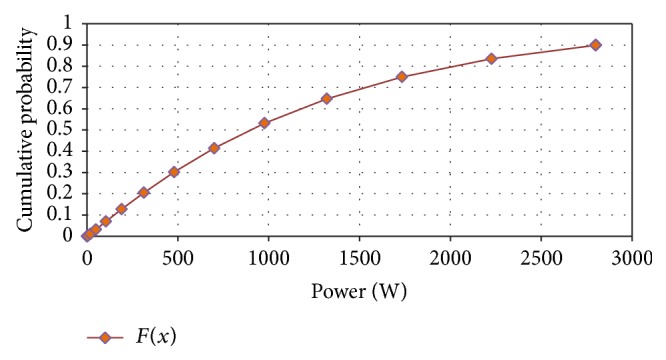
Unreliability versus power.

The reliability and unreliability prediction of the helical wind turbine depending on power was made based on experimental data. The parameters of the Weibull distribution were determined and the reliability and unreliability functions were calculated and graphically represented (Figures 9 and 10).

**Figure 11:**
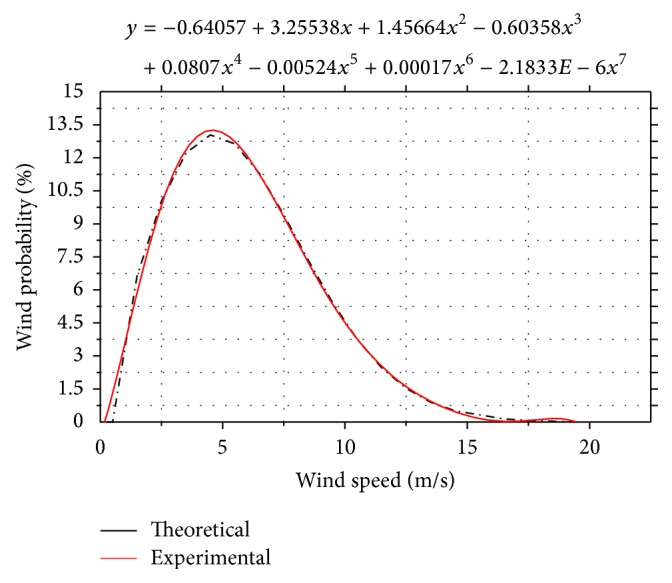
Wind probability at different wind speeds.

From the average wind speed per year it is possible to calculate the Weibull distribution of wind probability and the power which is generated in a day, in a month, or in a year. With these data we can calculate the time of payback of investment and the cost in Euro/kWh of the energy generated.  Figure 11 shows the wind probability in percentage at different wind speeds.

**Figure 12:**
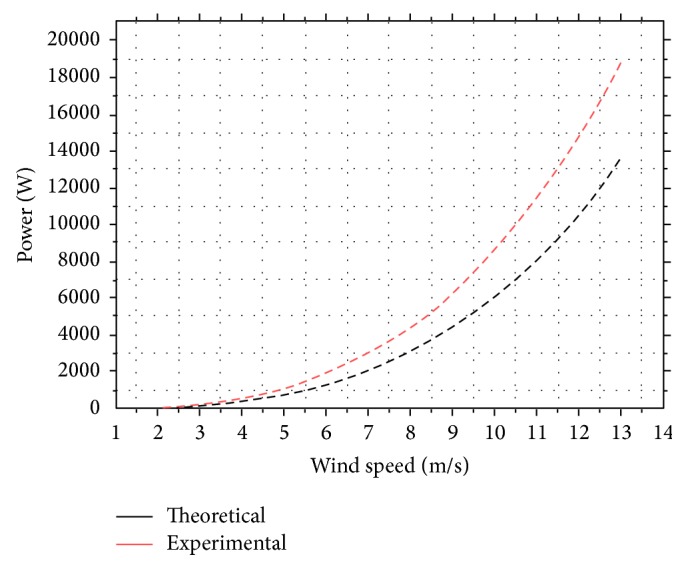
Power variation according to the wind speed. Comparison between experimental and simulation results.

A graphical representation of power variation according to the wind speed in the aerodynamic tunnel is illustrated in Figure 12.

The next values of energy were obtained according to the wind speed of 5.84 m/s in the IV-class.

- Daily energy output = 4.8 kWh.
- Annual energy output = 1.750 kWh.
- Monthly energy output = 143 kWh.
- Percent of operating time = 97.40%.

The results indicate that this type of wind turbine is very efficient at wind speeds between 3.5 and 7.5 m/s, when the probability of using it is between 9% and 13%. The analysis of experimental results indicates that this type of wind turbine at low wind speed is more efficient than other wind turbines with horizontal axis and with three blades.

## 4. Conclusions

As it has been proved by a lot of researchers and energy engineers, it will be a very positive and economic solution to replace the bigger part of diesel generators with stand-alone hybrid energy systems, especially in medium and high wind and solar potential locations.

The application of the Monte Carlo simulation allows us to determine the cumulative distribution curve and it helps to estimate the probability to obtain the daily and annual energy outputs with a specified wind speed. These charts can be used to establish upper and lower specification limits on energy production and this information can be very useful to optimize the parameter and components of wind turbine.

Based on simulation principles, the statistical processing of experimental data was performed. By this means, the wind speed, the output power, and the annual energy output were determined. Considering these parameters, the reliability function, unreliability functions, and failure rate were estimated. So, the reliability modeling and analysis of the wind turbine permit us to understand and minimize wind turbine operation and maintenance costs. Information derived from these measurements can help to identify where the problems are. Further interpretation of the data could help to optimize the wind turbine and it could assess if the optimum parameters of the system have been chosen.

A comparative analysis between the efficiency of our designed and tested wind turbine and different types of wind turbines [26–28], from point of view of output power and the percent of operating time, indicates that our experimental results for this type of wind turbine at low wind speed are more efficient than other wind turbines with horizontal axis.

The tests results have demonstrated the advantages of this type of vertical axis wind turbine, against the other types of wind turbines. In the time that will follow, based on the tests in laboratory and in aerodynamic tunnel, we intend to improve its performances.

## Figures and Tables

**Table 1:** Technical characteristics of wind turbine with vertical axis.

Power	1000 W
Wind speed	Cut-in wind speed: 1.8 m/s Nominal wind speed: 12 m/s Cut-out wind speed: 20 m/s
Blade numbers	2
Rotor diameter	1.20 m
Rotor height wind turbine with vertical axis	1.20 m
Current generator	GL-PMG 1000
Turbine weight	135 kg

**Table 2:** Simulation process results.

Parameter	Probability {*x* ≤ *x*_0_}	Probability {*x* ≤ *x*_1_}	Probability {*x* > *x*_1_}
Wind speed [m/s]	0.254	0.904	0.096
Daily energy output [kWh]	0.147	0.972	0.028
Annual energy output [kWh]	0.058	0.895	0.105
